# Assessing brain neuroplasticity: Surface morphometric analysis of cortical changes induced by Quadrato motor training

**DOI:** 10.1111/joa.14104

**Published:** 2024-06-26

**Authors:** F. Spani, F. Carducci, C. Piervincenzi, T. D. Ben‐Soussan, C. A. Mallio, C. C. Quattrocchi

**Affiliations:** ^1^ Department of Science and Technology for Sustainable Development and One Health Università Campus Bio‐Medico di Roma Rome Italy; ^2^ Neuroimaging Laboratory, Department of Physiology and Pharmacology Sapienza University of Rome (IT) Rome Italy; ^3^ Department of Human Neurosciences Sapienza University Rome Italy; ^4^ Research Institute for Neuroscience, Education and Didactics (RINED), Patrizio Paoletti Foundation Assisi Italy; ^5^ Department of Medicine and Surgery, Research Unit of Diagnostic Imaging Università Campus Bio‐Medico di Roma Rome Italy; ^6^ Fondazione Policlinico Universitario Campus Bio‐Medico, Operative Research Unit of Diagnostic Imaging and Interventional Radiology Rome Italy; ^7^ Centre for Medical Sciences‐CISMed University of Trento Trento Italy

**Keywords:** 3D brain cortex, generalized procrustes surface analysis, magnetic resonance imaging, mindfulness, sensorimotor training, shape variation

## Abstract

Morphological markers for brain plasticity are still lacking and their findings are challenged by the extreme variability of cortical brain surface. Trying to overcome the “correspondence problem,” we applied a landmark‐free method (the generalized procrustes surface analysis (GPSA)) for investigating the shape variation of cortical surface in a group of 40 healthy volunteers (i.e., the practice group) subjected to daily motor training known as Quadrato motor training (QMT). QMT is a sensorimotor walking meditation that aims at balancing body, cognition, and emotion. More specifically, QMT requires coordination and attention and consists of moving in one of three possible directions on corners of a 50 × 50 cm^2^. Brain magnetic resonance images (MRIs) of practice group (acquired at baseline, as well as after 6 and 12 weeks of QMT), were 3D reconstructed and here compared with brain MRIs of six more volunteers never practicing the QMT (naïve group). Cortical regions mostly affected by morphological variations were visualized on a 3D average color‐scaled brain surface indicating from higher (red) to lower (blue) levels of variation. Cortical regions interested in most of the shape variations were as follows: (1) the supplementary motor cortex; (2) the inferior frontal gyrus (*pars opercolaris*) and the anterior insula; (3) the visual cortex; (4) the inferior parietal lobule (supramarginal gyrus and angular gyrus). Our results show that surface morphometric analysis (i.e., GPSA) can be applied to assess brain neuroplasticity processes, such as those stimulated by QMT.

## Graphical abstract

Morphological markers for brain plasticity are still lacking, and their findings are challenged by cortical brain surface variability. To overcome the “correspondence problem,” we used a landmark‐free method (generalized Procrustes surface analysis, GPSA) to investigate cortical surface shape variation in healthy volunteers practicing Quadrato motor training (QMT), a sensorimotor walking meditation. MRIs of the practice group (baseline, 6, and 12 weeks of QMT) were compared to non‐practicing volunteers. Significant morphological variations were found in the supplementary motor cortex, inferior frontal gyrus, anterior insula, visual cortex, and inferior parietal lobule. Results show GPSA can assess neuroplasticity processes stimulated by QMT.

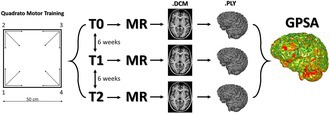

## 1 INTRODUCTION

Recent studies on the shape of the brain in vertebrates have made significant advancements, utilizing cutting‐edge technologies like magnetic resonance imaging (MRI), computerized tomography (CT) scans, and advanced computational modeling (Hoops et al., 2018; Navarrete et al., 2018; Witmer et al., 2003). These technologies have enabled researchers to examine the intricate details of brain morphology with unprecedented precision. One key area of focus has been the comparison of brain structures across different vertebrate species to understand the evolutionary adaptations and functional specializations. For instance, research has highlighted how the expansion and folding patterns of the cerebral cortex vary among mammals, reflecting differences in cognitive abilities and sensory processing (an Essen et al., 2018; van Essen et al., 2019). Additionally, studies have explored the relationship between brain shape and ecological factors. For example, variations in the size and shape of the hippocampus have been linked to spatial navigation capabilities in birds and mammals. This indicates that environmental demands can significantly influence brain morphology (Rolls & Wirth, 2018). Advancements in genomics have also shed light on the genetic underpinnings of brain development and shape. By comparing the genomes of different species, scientists have identified specific genes that contribute to the development of unique brain structures (Leung et al., 2022). Overall, the state‐of‐the‐art in vertebrate brain morphology studies is marked by a multidisciplinary approach, integrating anatomical, ecological, and genetic perspectives (Healy & Rowe, 2007). This holistic view provides deeper insights into the complexity and diversity of brain evolution and function across the vertebrate lineage (Striedter, 2005).

The research of biological markers for human brain diseases (including mental illness) is still a challenge, especially when it comes to brain images, mostly due to the extreme variation of human brain anatomy (Klein et al., 2017). Among bio‐markers, morphological ones seem to show more accuracy in predicting the prognosis of patients with behavioral disorders than behavioral scales or structured interviews ((Dagum, 2019; Klein et al., 2017) and references therein).

MRI have proven that brain plasticity occurs not only under pathological states (i.e., “negative” neuroplasticity processes) but also after motor training, mental training, and mindfulness (i.e., “positive” neuroplasticity processes) (Baglio et al., 2021; Eskildsen et al., 2013; Teixeira‐Machado et al., 2019; Valk et al., 2017; Wee et al., 2013). However, the wide shape variation of the brain surface among human subjects hinders the accuracy of any measurement or landmark‐based analysis, generating what is known as the “correspondence problem.” According to Klein and colleagues (Klein et al., 2017), in order to compare measures collected on brain images, it is mandatory to firstly set either a correspondence or mapping for each scanned brain, overcoming the standard co‐registration methods. In fact, image co‐registration, commonly used in neuroimaging by brain template or labeled atlas, does not always guarantee the exact spatial correspondence (Crum et al., 2005) as it forces each subject's brain shape to fit within a single template that could not be representative of the entire studied group. Nevertheless, several brain imaging studies used co‐registration for investigating image similarity and comparing brains either in terms of rectilinear volume (Ritchie et al., 2015) or by approximating them to spheres (Bansal et al., 2012).

To compare different features within or among groups in brain imaging, the most used methods for quantification are as follows: (1) the characterization of both quantities and grey‐scale value distribution within a volume; (2) the assessment of similarity and dissimilarity between a given co‐registered brain with a standard one; (3) the direct measurement of shape intended as all geometrical information remaining after having performed a superimposition (i.e., to “superimpose” one or more objects means to optimally translate, to uniformly scale and to rotate them allowing the evaluation of shape differences) (Dryden & Mardia, 1998). Volume, surface area, and cortical thickness of limited regions are shape measurements available in online brain image datasets that may provide more sensitive and specific morphological markers, thus significantly improving results if combined into multivariate analyses (Ecker et al., 2010).

Very few studies have explored shape variation of brain surface by using the identification of discrete homologous landmarks (i.e., discrete anatomical loci found in all samples) that are fundamental to perform a geometric morphometric analysis. They focused mostly on disposition of sulci (Free et al., 2001), subcortical structures (Bookstein et al., 2001), and evolutionary patterns (Bruner, 2004). In order to overcome difficulties due to the extreme morphological complexity of brain cortical surface, associated with difficulties in the application of landmark‐based techniques (i.e., geometric morphometrics, GM), we applied the generalized procrustes surface analysis (GPSA, (Pomidor et al., 2016)). The GPSA adapts the iterative closest point (ICP, (Shi et al., 2019; Shi et al., 2020)) family of algorithms to the generalized procrustes analysis (GPA, (Rohlf & Slice, 1990)) paradigm, and introduces a new metric for quantification of shape differences between two surfaces. It is a continuous variable, known as the procrustes surface metric (PSM), summarizing shape variation, in turn, by an automated shape analysis on scanned brains that uses the nearest neighbor pairings from the final superimposition to define the homology between analyzed surfaces (i.e., without homologous landmark assistance). One of the strengths of analyses performed in a point‐wise manner, considering the whole surface rather than some manually chosen landmarks, is the large amount of data that is available for analysis. The GPSA was previously applied for investigating shape variation of other biological structures, most of them were “solid” structures such as gastropod shells (Harvey et al., 2018), vertebrate pelvis (Santos‐Santos et al., 2019), and vertebrate skulls (Dos Reis et al., 2021; Fruciano et al., 2017; Gonzalez, 2019; Pomidor et al., 2016; Sookias, 2020; Wasiljew et al., 2021). To date, such methodology was successfully applied to “soft” anatomical structures in a single case study on squamate brains (Macrì et al., 2019) revealing a variation of cerebellar architecture linked to different locomotor specializations.

Among all “positive” neuroplasticity processes arising after motor training, this study focuses most on effects of QMT on cortical plasticity. QMT is a new whole‐body mindful practice that aims at improving attention, coordination, creativity, and mindfulness (Ben‐Soussan et al., 2013; Ben‐Soussan, Berkovich‐Ohana, et al., 2014). QMT was consistently found to enhance cognitive flexibility, spatial cognition, and neuroplasticity (for recent reviews see (de Fano & Leshem, 2019)).

As the result of a current running project named MOTOBRAIN, previous studies highlighted behavioral (Ben‐Soussan, 2014; Ben‐Soussan et al., 2013; Ben‐Soussan, Berkovich‐Ohana, et al., 2015), neurophysiological (Ben‐Soussan et al., 2013; Ben‐Soussan, Avirame, et al., 2014; Ben‐Soussan, Glicksohn, & Berkovich‐Ohana, 2015), and neuroanatomical changes (Piervincenzi et al., 2017) focused on white matter after QMT; however, none of them focused on shape variation of the cortical surface.

The present study aims at assessing for the first time the performance of GPSA on evaluating brain surface shape changes of a group of volunteers longitudinally examined before and after regularly practicing the QMT and comparing it with a group of volunteers who did not undergo the QMT. As a secondary aim, we explored potential gender‐wise differences in brain shape variations related to the practice of QMT.

## 2 METHODS

The present study is based on a sub‐sample of volunteers (see (Lasaponara et al., 2016) for more details) originally involved in a larger project aimed at investigating the longitudinal effect of QMT using different brain imaging techniques. Complete experimental procedures were analyzed and discussed elsewhere (Lasaponara et al., 2017; Piervincenzi et al., 2017).

### 2.1 The Quadrato motor training (QMT)

**FIGURE 1:**
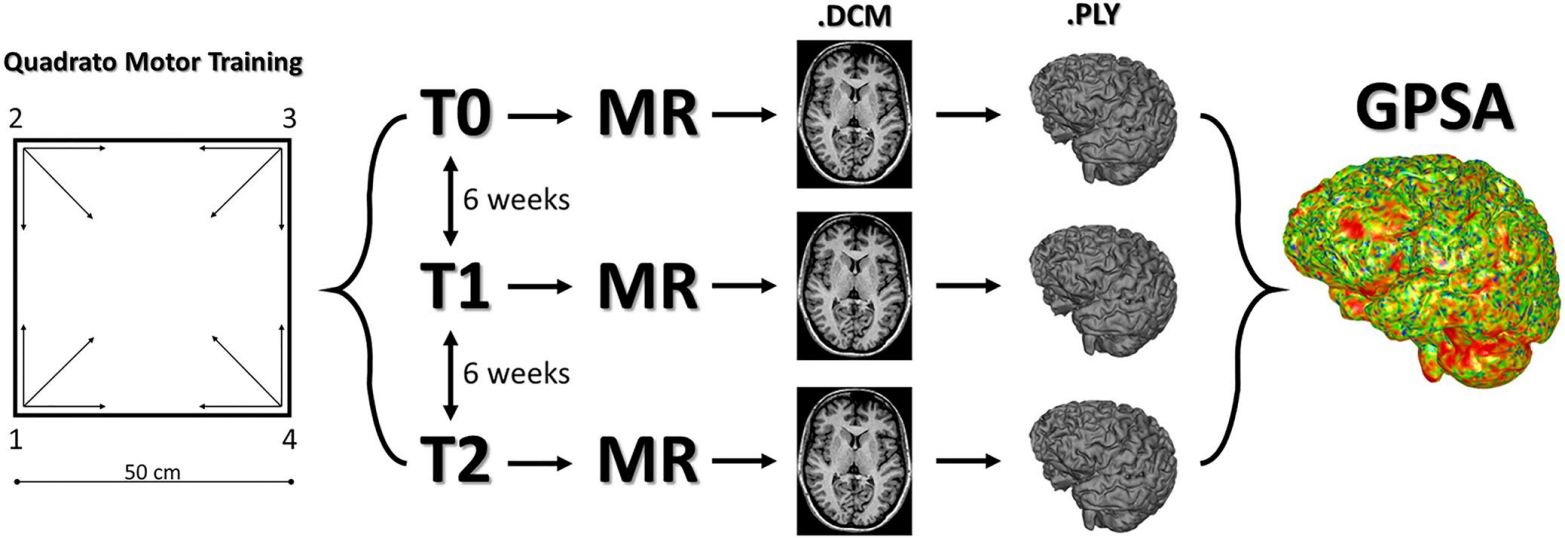
The Quadrato motor training (QMT) graphical illustration (left) followed by the experiment flowchart (right). QMT: following a sequence of 69 vocal commands indicating both the start and end corners to which the patient must move. Only one‐step movements are allowed, for a total of 12 movements. Experiment flowchart (for both practice group of QMT and naïve group): brain magnetic resonance images of both groups (46 volunteers) were acquired at each time point (t0, t1, and t2) every 6 weeks, and manually segmented for 3D visualization of brain volumes. The generalized procrustes surface analysis was performed in several rounds.

The QMT (created by Patrizio Paoletti, (Ben‐Soussan & Paoletti, 2014)) is a sensorimotor training based on a 0.5 m × 0.5 m square with a number from 1 to 4 at each corner (Figure 1). The daily training consisted of an audible sequence of 69 recorded commands indicating both the start and the end corner to which the patient should move (e.g., “one‐two” means a move from corner 1 to corner 2), lasting a total of 7 min, with a movement sequence paced at a rate of an average of 0.5 Hz (comparable to a slow walking rate). All movements were always in one step (3 directions × 4 corners = 12 single movements): 2 forward, 2 backward, 2 left, 2 right, and 4 diagonals. Participants were required to begin all movements with the leg closest to the center of the square, keep eyes focused straight ahead and hands loose at the side of the body, immediately continue with the next instruction, and never stop in case of mistakes.

### 2.2 Subjects and procedure

We investigated MRI of the brain of 40 healthy volunteers (i.e., 23 women and 17 men, mean age ± SD: 35 ± 5 and 36 ± 6, respectively) undergoing 3 months of daily QMT and hereafter referred to as ‘practice group’. In addition, brain MRI of six healthy volunteers (i.e., 3 women and 3 men, mean age ± SD: 27 ± 1 and 25 ± 1, respectively) who never experienced QMT, were acquired as “naïve group.” Imaging data were acquired using a Siemens 1.5 T MAGNETOM Avanto (Siemens, Erlangen, Germany) whole‐body scanner equipped with a 12‐element Head Matrix coil. A sagittal magnetization‐prepared rapid acquisition gradient echo (MPRAGE) t1‐weighted sequence (TR = 2400 ms, TE = 3.61 ms, TI = 1000 ms, flip angle = 15°, FOV = 240 mm × 240 mm, NEX = 1, matrix = 192 × 192, 1.00 × 1.00 mm2 in‐plane resolution, horizontal slices with slice thickness of 1.2 mm and no gap) was used to acquire and collect brain structural images.

Following both inclusion and exclusion criteria reported in Table 1, a total of 46 healthy volunteers were involved in the study. Forty of them underwent QMT as the practice group, while six of them did not and served as the control group. The longitudinal protocol applied to both practice and naïve group consisted of three time points (t0, t1, and t2) temporally spaced every 6 weeks (see Figure 1 for the experiment flowchart).

**TABLE 1:** Eligibility criteria for participants.

Inclusion criteria
Age between 18 and 45 years
Right‐handedness
No motor, emotional, cognitive, or developmental coordination disorders
No previous practice of the QMT or other motor activation programs
Exclusion criteria
History of traumatic injury, previous neurosurgery, stroke, inflammatory/infective brain disease
Co‐morbidity of congenital metabolic diseases or malformations
Diagnosis of one histologically proven primary cancer (<1 year)
Vitamin B12 deficiency, positive serology for secondary dementia (RPR/VDRL, HIV, anti‐Borrelia, abnormal thyroid function
Clinical evidence of depression or other psychiatric conditions, epilepsy, drugs, or alcohol addiction (according to DSM IV‐TR)
Severe cognitive impairment (Mini‐Mental State Examination ≤24)
Diagnosis of malnutrition
Chronic or acute inflammatory disease
Hearing or visual impairment or motor deficits incompatible with the workout
Hormone replacement therapy
Current or recent history of smoking (i.e., not smoking during the last year)
Lack of one out of three brain MRI

*Note*: Inclusion and exclusion criteria adopted in the present study for the inclusion of each volunteer in both practice (experimenting Quadrato motor training) and naïve group (never experimenting Quadrato motor training).

### 2.3 Ethics statement

After verifying that participants were able to understand, they were informed of all procedures. They were free to interrupt the QMT and drop out of the study at any time for any reason, without any prejudice. Written informed consents were obtained individually, according to the declaration of Helsinki. The ethical committee of the Università Campus Bio‐Medico di Roma (Rome – IT) approved the experimental phase I of the study entitled “Effect of quadrato MOtor Training On the BRAIN of healthy volunteers” (MOTO‐BRAIN, 09/14 PAR ComEt CBM). Also, the experimental protocol was approved by the ethical committee of the Università Campus Bio‐Medico di Roma (Rome – IT). Compliance with GCP (good clinical practice) was warranted and data were collected following the ALCOA (Attributable, Legible, Contemporaneous, Original, and Accurate) algorithm. The TREND checklist was also accomplished (see Table S1).

### 2.4 Manual segmentation procedure

The DICOM data from all 138 MPRAGE images of the 46 participants were individually loaded into Amira (ver. 5.4.5). Employing the “Isosurface” module was applied, which encloses all parts of a volume that are brighter than some user‐defined threshold, for providing an impression of the 3D shape of an object. For each 3D brain scan data, a threshold value ranging overall between 180 and 300 was set in the “properties” menu, which determines the value for isosurface computation. The slider is automatically adjusted to cover the whole range of data values and the operator can choose the most suitable value for visualizing the 3D shape (i.e., brain shape) of the object. The visualized 3D surface of the entire head, comprehensive of the brain, was extracted with the “extract surface” module. The researcher (FS) performing manual thresholding was blinded to the group and to the time point membership of each scan, in order to avoid the introduction of any experimental bias. To virtually isolate only the brain surface, the “surface editor” option, available in the properties panel, allows to modify a triangular surface in several ways, for example, by removing or refining triangles, by flipping edges or moving points. In this case, triangles belonging to other anatomical parts surrounding the brain were manually selected and deleted. At the end of the manual segmentation procedure, the virtually extracted 3D brain meshes were saved as a 3D polygon volumetric model (.ply), usually used for storing points and geometries. The total time needed to extract and visualize 3D brain meshes was, on average, 20 min per MRI scan.

### 2.5 Post‐processing and 3D rendering procedure

Polygon models were then post‐processed with Geomagic Studio 2014 software. The extracted surface, loaded into Geomagic Studio 2014, was corrected by deleting (automatically and manually) computational errors in the mesh such as non‐manifold edges, self‐intersections, highly creased edges, spikes, small components, small tunnels, small holes. Only the external surface of the brain was kept, by emptied 3D models, to avoid anatomical non‐homologies in the brain's internal structure. The total number of polygons was fixed to 170,000 and the total time needed to obtain meshes suitable for GPSA analyses was, on average, 10 min.

### 2.6 The GPSA

The free and open‐source GPSA software was downloaded from the lab website (URL: http://morphlab.sc.fsu.edu/index.html, accessed June 14, 2021). To run GPSA, the first action is the prototype selection among 3D scanned brains, in order to allow the superimposition of individuals in the dataset. This surface, as the authors of GSPA method suggested (Pomidor et al., 2016), should be the most complete, most representative, least morphometrically atypical individual in the sample. For this reason, a first GPSA was performed on a subset of brain surfaces collecting all t0 surfaces, belonging to both practice and naïve group (since the formers never experienced the QMT at t0 and are then considered equal to the naïve group). An average surface of all scanned brains at t0 time (acronym = t0_mean) was then calculated (among all superimposed shapes) and used as the prototype surface for subsequent shape analyses. In fact, GPSA uses surface scans, and their polygon vertex coordinates to be homologized, thus performing superimposition to the prototype surface (i.e., “superimposing” two objects by optimally translating, uniformly scaling, and rotating them allowed evaluation of shape differences) via symmetric ICP. Prototype pairings were used to update the average position for individual points on the prototype surface and to remove duplicate points. Then, shape distance metrics (i.e., PSMs) analogous to Procrustes distances (used to quantify similarity or dissimilarity of 3D shapes) were calculated to measure the difference between superimposed surfaces. This metric uses the nearest pairings from the final superimposition to define the homology between surfaces. Considering surfaces A and B, where *p* and *q* represent a point and its nearest neighbor and m is the number of points on the surface, this metric is as follows:

$$D=\sqrt{\frac{1}{2m_{A}}\sum_{i=1}^{m_{A}}{\left(p_{A,i}-q_{B,i}\right)}^{2}+\frac{1}{2m_{B}}\sum_{i=1}^{m_{B}}{\left(p_{B,i}-q_{A,i}\right)}^{2}}$$

This metric is similar to a root‐mean‐square distance, but it uses the points from both surfaces and weights each surface appropriately so that they each contribute equally to the metric. The relation between this metric and Procrustes distance *P* is simply:

$$D=\sqrt{\frac{1}{m}}.P$$

Scanned brain surfaces were grouped into different subsets (see Table 2), according to the purpose of each analysis, and several GPSAs were carried on (i.e., one for each subset). Four main rounds of GPSA were executed. The first round involved the naïve group, to highlight (or not) any shape variation on the cortex. We executed two GPSA sessions: (1) on all t1 brain meshes (CTR_t1); (2) on all t2 brain meshes (CTR_t2). The second round involved the practice group, to focus on shape variation affecting specific cortical regions after 6 (i.e., t1) and 12 (i.e., t2) weeks of QMT. We executed two GPSA sessions as well: (1) on all t1 brain meshes (MB_t1); (2) on all t2 brain meshes (MB_t2). The third round involved gender‐wise GPSAs, to focus on shape variation affecting specific cortical regions in females and males (separately) after 6 (i.e., t1) and 12 (i.e., t2) weeks of QMT. We executed four GPSA sessions as well: (1) on all t1 female brain meshes (MB_t1_F); (2) on all t2 female brain meshes (MB_t2_F); (3) on all t1 male brain meshes (MB_t1_M); 4) on all t2 male brain meshes (MB_t2_M). To finally compare results of third round of GPSA with the naïve group, additional analyses (fourth round of GPSA) were performed on naïve group (i.e., four GPSA sessions) by separating them into four subsets: (1) t1 female brain meshes (CTR_t1_F); (2) t2 female brain meshes (CTR_t2_F); (3) t1 male brain meshes (CTR_t1_M); (4) t2 male brain meshes (CTR_t2_M).

**TABLE 2:** Subsets involved in generalized procrustes surface analysis.

T time	Group	Sex	Acronym
t1	Naïve	M + F	CTR_t1
t2	Naïve	M + F	CTR_t2
t1	Practice	M + F	MB_t1
t2	Practice	M + F	MB_t2
t1	Naïve	F	CTR_t1_F
t2	Naïve	F	CTR_t2_F
t1	Naïve	M	CTR_t1_M
t2	Naïve	M	CTR_t2_M
t1	Practice	F	MB_t1_F
t2	Practice	F	MB_t2_F
t1	Practice	M	MB_t1_M
t2	Practice	M	MB_t2_M

*Note*: Scanned brain surfaces were grouped into different subsets depending on purposes of different analyses. For each subset used to perform the GPSA, the time point (“T time” column, t1 = MRI scans acquired after 6 weeks, t2 = MRI scans acquired after 12 weeks), the membership group (“Group” column, “naïve” = volunteers never practiced QMT, “practice” = volunteers practiced QMT), the sex (F = female, M = male) and the acronym used for GPSA are given.

Point‐wise shape information was visualized over the mean surface resulting from the superimposition of all selected meshes at the end of each GPSA. The variance at every point on the mean surface was calculated and then mapped to colored vertices, to emphasize the regions mostly associated with shape variation: blue vertices standing for low values of variance (i.e., low shape variation), red vertices standing for high values of variance (i.e., high shape variation). Following this approach, we were able to generate a three‐dimensional heatmap representative of the subset variation. All the analyses were run into the GPSA software and the duration of each analysis depended on the number of meshes involved in the analysis itself (from few minutes to 15 min).

### 2.7 Cortical thickness assessment

All participants were processed with the Computational Anatomy Toolbox 12 (CAT12) toolbox (http://www.neuro.uni‐jena.de/cat/, version r1742) within SPM12 (http://www.fil.ion.ucl.ac.uk/spm/software/spm12/, version 7771) using MATLAB (R2022b) for automatic measurement of cortical thickness surfaces at three timepoints (t0, t1, and t2). According to the standard longitudinal protocol (http://www.neuro.uni‐jena.de/cat12/CAT12‐Manual.pdf), default parameter settings were utilized for processing MR data. For further details see Supplementary Materials. The resulting cortical thickness surfaces were qualitatively compared with GPSA heatmaps.

### 2.8 Principal coordinate analysis

To summarize the description of shape variability that interested analyzed subsets, through a low number of descriptors, we ran an ordination analysis for dimension reduction known as principal coordinate analysis (PCOORD; (Gower, 1966)) after a last round of GPSA. This last GPSA used the t0_mean brain meshes as prototype (i.e., the same meshes chosen for previous analyses), and the subsequent PCOORD analysis that involved only mean shapes resulting from previously executed GPSA (see above): t1 and t2 mean brain meshes of practice group; t1 and t2 mean brain meshes of naïve group. In brief, homologized vertex coordinates of selected mean brain meshes were regressed onto their scores of axes and this allowed to obtain both variances of the projections of the specimens on the PCOORD axes and the rate of variance accounted for by each axis (see (Pomidor et al., 2016) for more details).

## 3 RESULTS

### 3.1 GPSA on the naïve group

**FIGURE 2:**
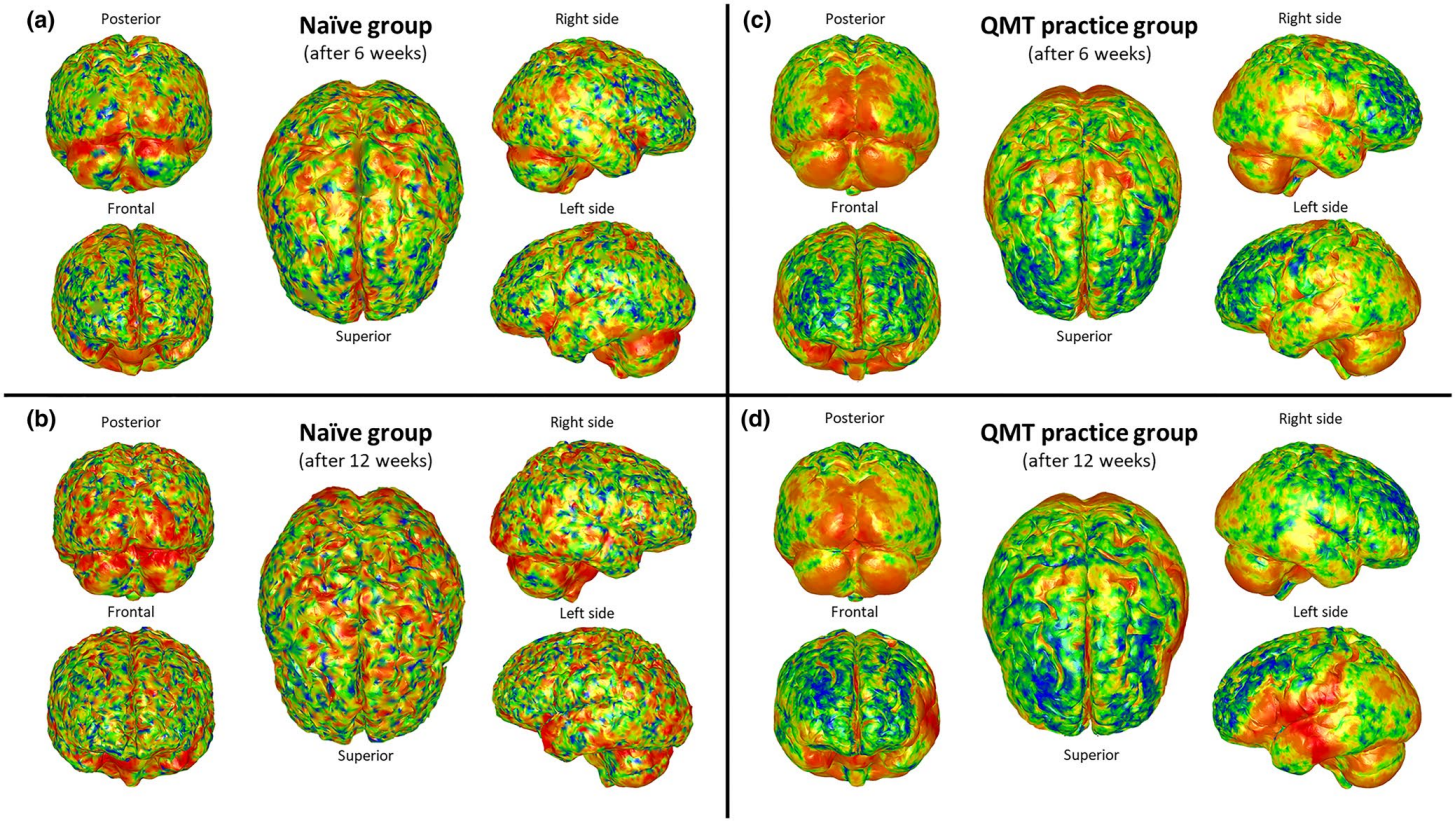
Heatmaps (i.e., morphological variation between two time points) of mean brain meshes of naïve (a, b) and practice groups (c, d), after 6 (a, c) and 12 weeks (b, d). The variance at every point on the mean surface was calculated and then mapped to colored vertices, to emphasize the regions mostly associated with shape variation: blue vertices standing for low values of variance (i.e., low shape variation), red vertices standing for high values of variance (i.e., high shape variation).

The first round of GPSA included two different analyses performed on both t1 and t2 brain meshes of the naïve group. Figure 2a shows the mean surface resulting from the GPSA on t1 brain meshes of the naïve group, with the heatmap of variance. These values are calculated from the covariance matrix of the set of nearest neighbor points for each point on the prototype. Figure 2b shows the heatmap of variance on the mean surface resulting from the GPSA of t2 brain meshes of the naïve group. In both heatmaps shown in Figure 2a,b, no cortical regions appear to be significantly highlighted, except for some punctual and sparse localizations without relevant morpho‐functional significance.

### 3.2 GPSA on practice group

The second round of GPSA included two additional analyses performed on both t1 and t2 brain meshes of the practice group. Figure 2c shows the mean surface resulting from the GPSA on t1 brain meshes of the practice group, with the heatmap of variance. After 6 weeks of QMT, most of shape variation (i.e., high values of variance, red‐colored regions) involves the visual cortex (Figure 2c, posterior view) and the supplementary motor cortex (Figure 2c, superior view). Minor changes were detected bilaterally as orange‐yellow colored in correspondence of the superior and inferior temporal gyrus, supramarginal gyrus, and angular gyrus (Figure 2c, right side view; Figure 2c, left side view). Figure 2d shows the mean surface resulting from the GPSA on t2 brain meshes of the practice group, with the heatmap of variance. After 12 weeks of QMT, most of red colored regions, indicating high shape variation (i.e., high values of variance) were located on the visual cortex (Figure 2d, posterior view), on the inferior frontal gyrus (*pars opercularis*) and anterior insula, and on the inferior parietal lobule (supramarginal gyrus and angular gyrus) (Figure 2d, left side view). Minor changes were detected on the superior side (Figure 2d, superior side view) and on the right side (Figure 2d, right side view), with the same color scale, in correspondence of the supplementary motor cortex and of superior temporal gyrus, respectively.

### 3.3 Gender‐wise GPSA

**FIGURE 3:**
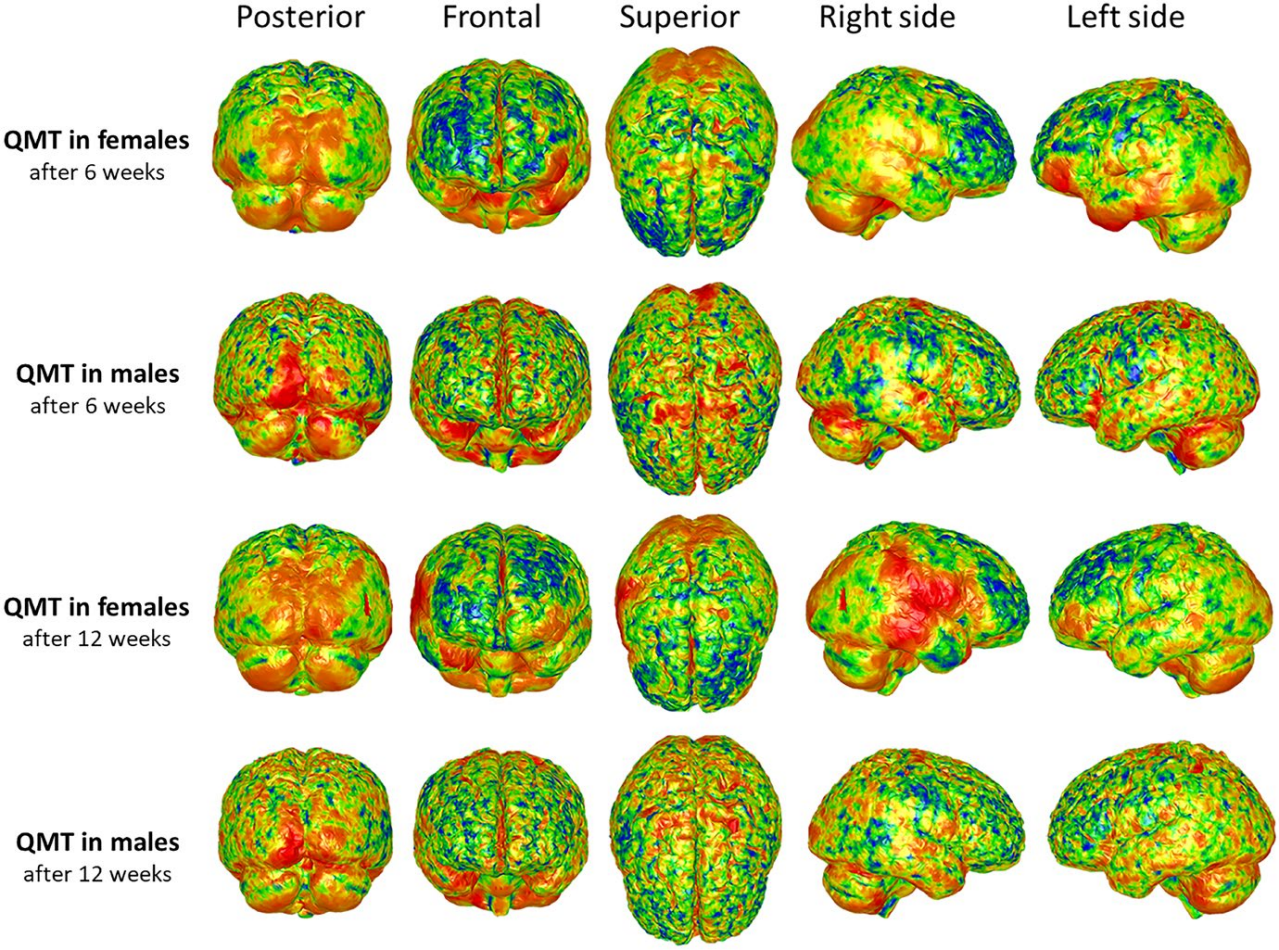
Heatmaps of the variance from the mean surface, used as prototype (and calculated as the average surface to which all T0 brain meshes in the sample are superimposed over the course of GPSA), for brain meshes of the practice group after 6 and 12 weeks, divided into females and males.

The third round of GPSA focused on any possible effect of QMT on brain cortex by also considering the gender variable. Comparing effects of QMT on cortex shape variation some differences appeared between females and males (see Figure 3). After 6 weeks of QMT, female cortical shape variation was mostly localized on the left *pars opercularis* and the temporal lobe. Minor variation was detected on the visual cortex. In males, after the same time of QMT, high levels of shape variation were focused on the visual cortex and the supplementary motor cortex. All previous cortical regions affected by shape variation after 6 weeks, and observed in both females and males, were confirmed after 12 weeks of QMT except for the *pars opercularis* and temporal lobe on the left side in females. In fact, the inferior frontal gyrus (*pars opercularis*), the anterior insula as well as parieto‐temporal areas on the right side of females (belonging to the practice group) appeared to be regions most affected by cortical shape variation after 12 weeks of QMT.

### 3.4 CAT 12 versus GPSA

**FIGURE 4:**
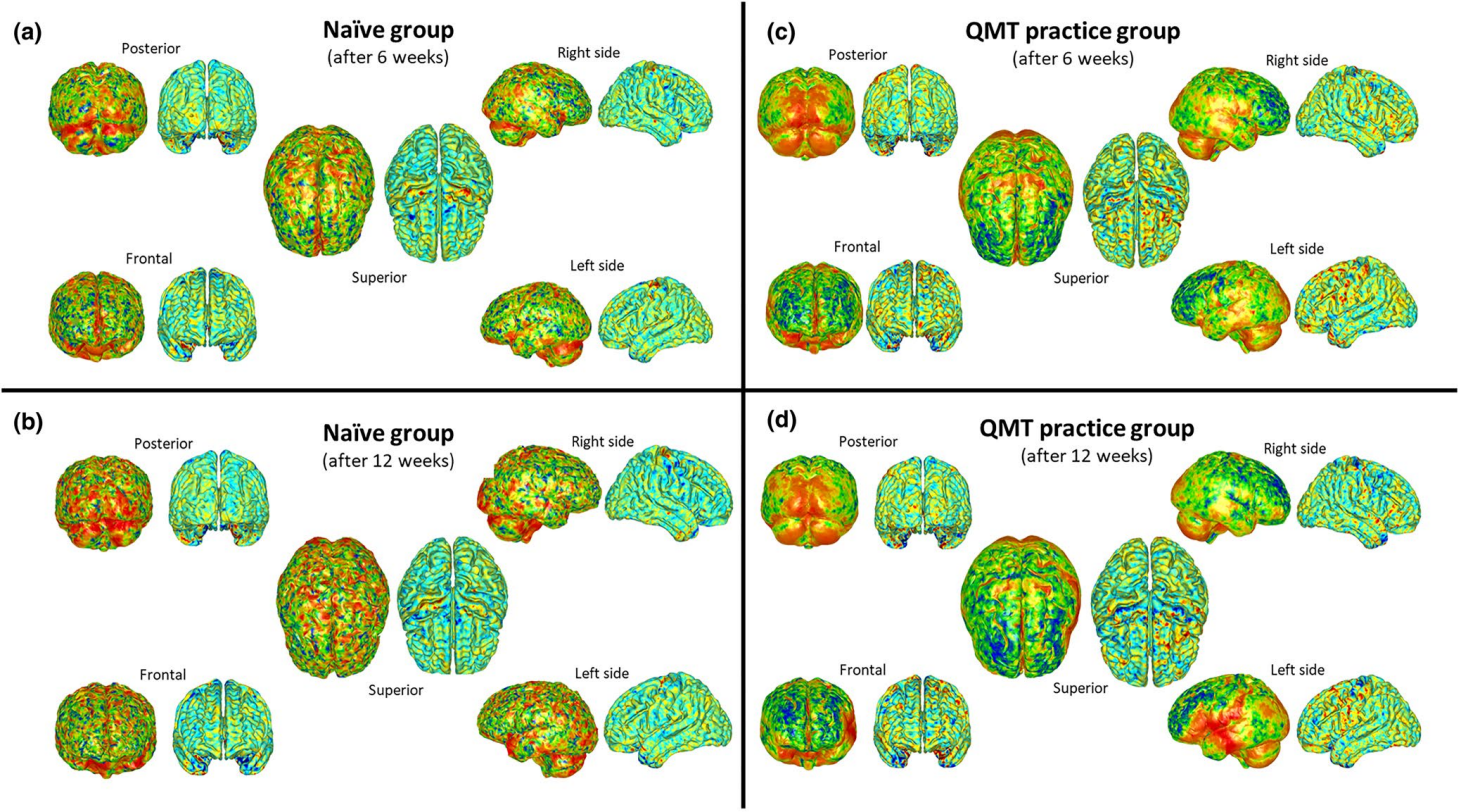
Comparisons between generalized Procrustes surface analysis heatmaps (calculated as the average surface to which all t0 brain meshes in the sample are superimposed over the course of GPSA; left meshes) and cortical thickness heatmaps (calculated as averaged‐resampled cortical thickness surfaces; right meshes) of mean brain meshes of naïve (a, b) and practice groups (c, d), after 6 (a, c) and 12 weeks (b, d).

Qualitative comparisons performed between cortical thickness surfaces and GPSA heatmaps seemed to show similar results with an increased sensitivity of GPSA in highlighting brain shape changes (for more details see Figure 4).

### 3.5 Principal coordinate analysis

**FIGURE 5:**
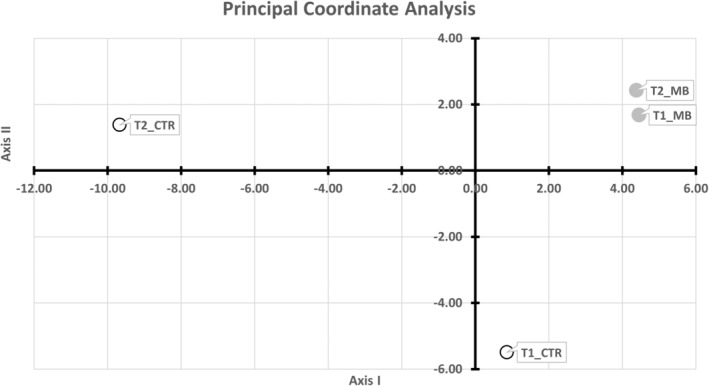
Principal coordinate analysis on all mean brain meshes. The geometric distances between mean brain meshes belonging to both naïve (t1_CTR and t2_CTR) and practice group (t1_MB and t2_MB) stand for the morphological diversity of the cortical shape shown in heatmaps (see Figure 2) existing in the naïve group and the practice group. The proportion of variance accounted for by each axis was 68% (axis I) and 21% (axis II).

Results of PCOORD are presented in Figure 5 with the rate of variance of shape accounted for by first (68%) and second (21%) axis. The spatial separation between mean brain meshes belonging to the naïve group, and mean brain meshes belonging to the practice group, confirmed the general morphological diversity of cortical shape which was shown above between the naïve and the practice group. At the same time, the proximity of t1 and t2 mean brain meshes validated previous results on shape variation observed on both t1 and t2 brain cortex which mainly interested supplementary motor cortex, the inferior frontal gyrus (*pars opercolaris*) and anterior insula, the inferior parietal lobule (supramarginal gyrus and angular gyrus) and visual cortex.

## 4 DISCUSSION

The major purpose of the present study is to propose, for the first time, results of a series of brain cortical surface analyses based on point‐by‐point distances (GPSA), therefore increasing the accuracy of detecting cortical changes to the level of shape variability. The GPSA was applied to two volunteers' groups, identified as practice group (experimenting the QMT for 12 weeks) and naïve group (the control group). Success of such analysis depends most on the resemblance to the reality of original brain meshes involved into the analyses. The main output of our analyses was the mean surface and the variance calculated at every point of the mean surface, allowing the creation of a 3D heatmap representative of the sample variation.

**FIGURE 6:**
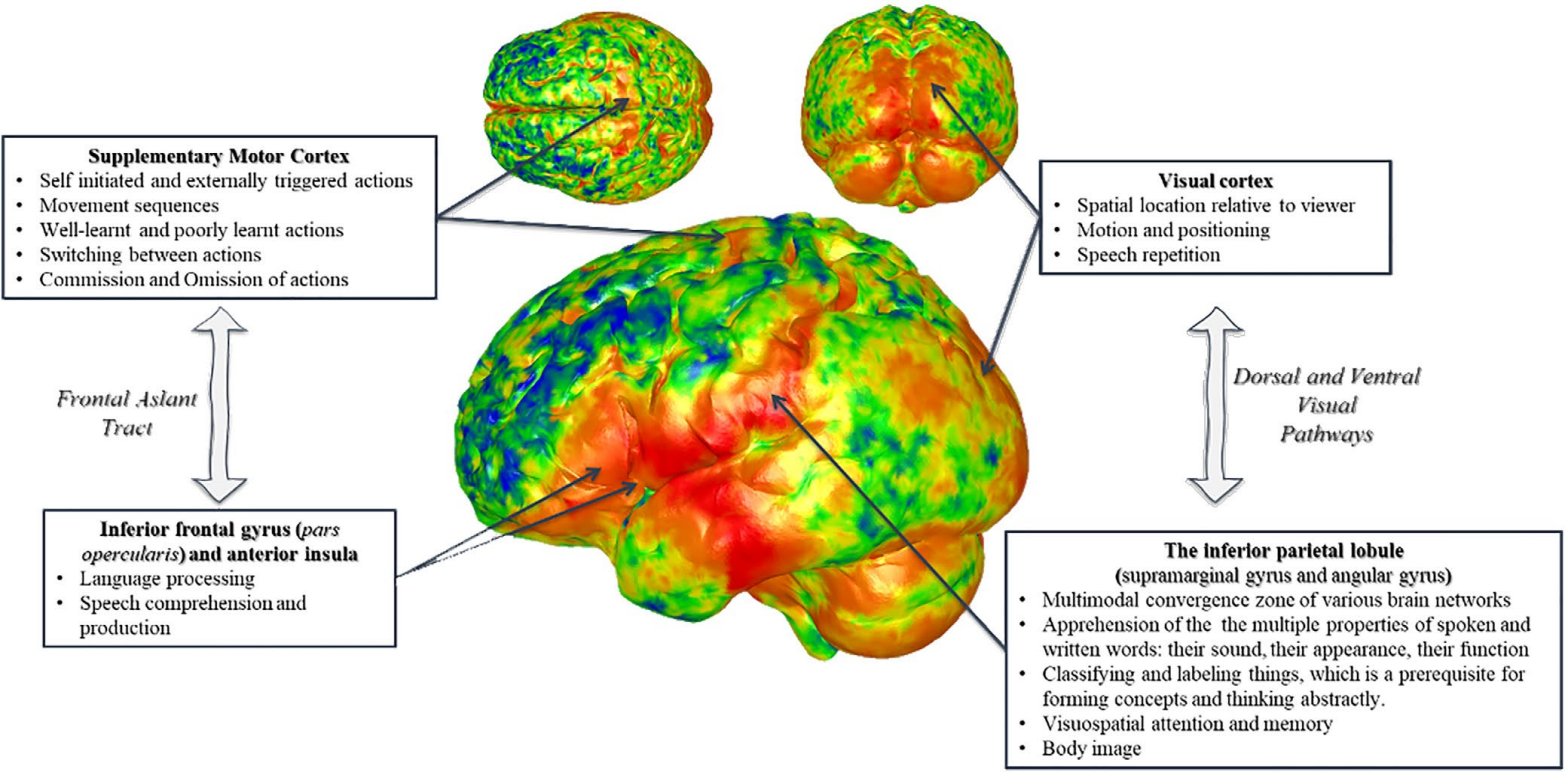
Graphical representation and summary diagram of obtained results of the present study. Main ‘hot spots’ of variation (red vertices standing for high values of variance, i.e., high shape variation) are displayed on an exemplary cortical brain mesh and linked to cortical regions showing the largest shape variation across the individuals in the different subgroups. Well‐known functions are listed for each area.

Summarizing the main results obtained by performing GPSA, “hot‐spots” of variation could be consistently displayed on both t1 and t2 average brain meshes of practice group cortical surfaces. That means that several localizations of the shape variation were identified as the average cumulative effect obtained from the individual comparison (and analysis) of all t1 and t2 brain meshes compared to t0 prototype brain meshes. Cortical regions showing the largest shape variation across the individuals in the different subgroups were as follows: (1) the supplementary motor cortex; (2) the inferior frontal gyrus (*pars opercolaris*) and the anterior insula; (3) the visual cortex; (4) the inferior parietal lobule (supramarginal gyrus and angular gyrus) (see Figure 6).

The structural connectivity between the supplementary motor cortex, the inferior frontal gyrus (IFG) and the anterior insula is supported by the frontal aslant tract (FAT, (Dick et al., 2019) and references therein) fiber pathway. If, on one hand, the IFG (including the “Broca's area”) is an important region for speech and language (Tremblay & Dick, 2016), on the other side, the selection and execution of movements in both speech and non‐speech domains mostly depend on supplementary motor area (SMA) and its connections with motor cortex (Côté et al., 2020; Russo et al., 2020). Available evidence demonstrated the connection between the left IFG and SMA in supporting the establishment of preferred motor response in the linguistic domain (Catani et al., 2013; Dick et al., 2019; Kinoshita et al., 2015). Precisely, our GPSAs on the practice group pointed out an intense morphological variation on the left hemisphere, affecting both motor cortex and the IFG.

The QMT practice and silence‐related experience were found to positively correlate with microstructural changes in the uncinate fasciculus (UF, (Ben‐Soussan et al., 2020)), a white matter tract which connects orbitofrontal cortex to the anterior temporal lobes (von der Heide et al., 2013) and is placed below the IFG and the anterior insula. Concerning UF functions, it is involved in multiple tasks such as naming, single‐word comprehension, response inhibition, face processing and monitoring of outcomes (Ben‐Soussan et al., 2020; Catani et al., 2013; Toller et al., 2022). On the other hand, disconnection of the uncinate fasciculus may cause reduced performance in memory tasks including temporally complex visual information (Gaffan & Wilson, 2008). Fridriksson and colleagues (Fridriksson et al., 2013) showed and proposed a role for the UF in maintaining as much as possible spared the auditory comprehension in patients with Broca's aphasia. Given that more functions are linked to all grey and white matter areas mentioned so far, in this context we could hypothesized that morphological variation observed in the brain cortex could be linked to one of those anatomical changes induced by QMT practice. Above all if it is taken into consideration the almost total absence of relevant changes in the naïve group.

The visual system in humans includes two separated pathways, a ventral stream that comprises areas of temporal cortex, and a dorsal stream that includes areas of posterior parietal cortex (Konen & Kastner, 2008). The former is usually involved into object vision, the latter into spatial vision and guided actions (Goodale & Milner, 1992; Ungerleider & Mishkin, 1982). Specifically, along the dorsal stream different regions could be activated if the subject is either viewing a movement or undergoing to a stationary stimulus (Grill‐Spector & Malach, 2004). In our analyses, the visual cortex resulted highly variable in its morphology after the experience of QMT for the practice group versus the naïve group. In fact, it is usually involved in functions also including the spatial location relative to viewer, the motion and positioning, and the speech repetition (Grill‐Spector & Malach, 2004). Finally, the parietal lobule (i.e., supramarginal gyrus and angular gyrus) showed high shape variation after the QMT practice. The inferior parietal lobule is known to be a multimodal convergence zone of various brain networks. Subsequently, it is involved in several functions such as the apprehension of the multiple properties of spoken and written words (e.g., their sound, their appearance, their function, etc.), classifying and labeling things (i.e., which is a prerequisite for forming concepts and thinking abstractly), visuospatial attention and memory, body image (Rizzolatti et al., 2006). These results are in line with previous results demonstrating improved spatial cognition following the QMT both in children and in adults (Ben‐Soussan, Berkovich‐Ohana, et al., 2014; Marson et al., 2021).

Unexpectedly we found a high cortical shape variation in t2 female subsets (i.e., female volunteers of the practice group after 12 weeks of QMT) detected on the right hemisphere, rather that the left (as observed in both the whole practice group and the male practice subset). In general, the functional brain asymmetry is a well‐known but poorly understood phenomenon suggesting that often language skills depend more upon left‐hemisphere activity whereas perception of spatial relationships depends primarily upon right‐hemisphere activity. In this context, another critical factor that complicates attempts to clarify the cerebral organization, and in particular the lateralization of visuospatial skills, is the gender (McGlone & Kertesz, 1973). Given that more studies had to be carried on concerning all morphological variations highlighted on the brain cortex, as well as the gender differences arisen, a possible explanation of our results could rely on neural differences in language processing existing between males and females, as observed by Baxter and colleagues (Baxter et al., 2003). They examined functional MRI patterns of 10 females and 9 males during a semantic processing task (i.e., semantic language skills refer to an understanding and appropriate use of meaning in single words, phrases, sentences, and even longer units). Both groups displayed activation of left inferior frontal gyrus, left superior temporal gyrus, and cingulate. Females only showed bilateral inferior frontal gyrus and superior temporal gyrus activation. However, further analyses revealed females had less diffuse left activation and greater right posterior temporal and insula region activation than males.

Despite several software solutions and algorithms are available for performing either automated or semi‐automated segmentation of brain, the present study suggests that a manual segmentation method, such as the one here applied, should be preferred to other automatic segmentation techniques: the manual segmentation of brain MRI, despite being time‐consuming, ensure the closeness of resulting 3D brain meshes to the real shape of scanned brain, avoiding any possible dummy shape variation due to automatic segmentation logarithms rather than effective brain cortical shape variation. At the same time, any artifact from scanning, extraneous anatomy, or missing data (holes) can introduce more errors to the analysis, so care must be taken to make sure any such issues are eliminated in the rendering phase. Then, the loss of point issues during iteration should be minimized by intraindividual analysis with an expected high level of homology.

Limitations of the study are mainly due to the applied methodology (i.e., GPSA), as this does not allow (at the current state of knowledge) to user‐friendly quantify the morphological variation point‐by‐point, but only to summarize it in a qualitative way (i.e., as displayed with heatmap). Moreover, the PCOORD analysis could be performed also on the entire brain surface group (and not only on mean surfaces, as performed here), but the morphological variation affecting each brain surface would hide differences between naïve and practice group. Additionally, the naïve group was recruited and scanned separately from the practice group; despite the same scanner and protocol were used we cannot exclude the confounder effect of time‐dependent variables, such as the gradient coil deterioration.

While QMT has shown promise in improving cognitive performance in humans, its feasibility in non‐human vertebrate species presents both opportunities and challenges. Many non‐human vertebrates, especially higher mammals such as primates and certain bird species like corvids and parrots, exhibit considerable behavioral flexibility and learning abilities (Bond et al., 2007). These species can learn complex sequences and tasks, making them potential candidates for adapting QMT. For example, studies have demonstrated that primates can learn and perform tasks involving sequences of movements (Kumpan et al., 2022; Ohbayashi, 2021), suggesting they could potentially be trained in QMT sequences. Successful implementation of QMT in non‐human vertebrates would require motivation through positive reinforcement techniques, such as food rewards or social reinforcement. Ensuring consistent and appropriate reinforcement schedules would be crucial to maintain engagement and learning. The cognitive capabilities of the species in question must align with the demands of QMT. Species with advanced motor control and spatial awareness, such as certain mammals and birds (Butler & Cotterill, 2006), would be more suited to QMT. Physical capabilities, including dexterity and the ability to perform precise movements, are also critical. Species with fine motor skills, like primates, would find it easier to perform the required sequences.

Ethical considerations are crucial when training non‐human vertebrates to ensure the training does not cause stress or harm and enriches their environment. Welfare standards should keep sessions short, engaging, and beneficial. Rigorous experimental designs with control groups, proper sample sizes, and longitudinal studies are necessary to assess QMT's efficacy. Adaptations are needed to fit the anatomical and cognitive traits of different species, as movements natural for humans may not be suitable for other animals. Effective communication through visual and physical cues is essential, and cognitive benefits should be assessed through indirect measures like behavior changes and neurobiological assessments. Despite these challenges, QMT can reduce boredom and stress in captive animals, improve cognitive functions, and offer insights into cognition and brain plasticity. With proper adaptations and controls, QMT could significantly benefit scientific research and animal welfare.

## 5 CONCLUSION

In our study, the GPSA proved to be extremely sensitive and highly feasible for human brain surface shape analysis, allowing to evaluate longitudinal modifications of cortex morphology either in healthy patients (as this study has shown) or pathological patients (future perspectives). Further implementations will regard quantitative comparisons with existing methods of cortical surface analysis, as well as correlative analyses with psychological data to reach the objective to monitor cortical shape variations at the individual level and infer on their relationship with clinical as well as behavioral correlates.

## AUTHOR CONTRIBUTIONS

TDBS and CCQ were responsible for the conceptualization. FS, CAM, and CCQ handled the data curation. FS conducted the formal analysis and developed the software. FC, TDBS, CAM, and CCQ carried out the investigation. FS and CCQ developed the methodology. CCQ oversaw the project administration, provided resources, and conducted the supervision. FS and TDBS created the visualization. FS and CCQ wrote the original draft. FS, FC, CP, TDBS, CAM, and CCQ contributed to the writing—review. FS and CCQ performed the editing in writing.

## FUNDING INFORMATION

The present study is part of the project “Effects of quadrato MOtor Training On the BRAIN of healthy volunteers—MOTOBRAIN” (MOTO‐BRAIN, 09/14 PAR ComEt CBM) funded by Patrizio Paoletti Foundation.

## CONFLICT OF INTEREST STATEMENT

None.

## CONSENT

After having verified sufficient understanding, participants were informed of all procedures. They were free to interrupt the QMT and drop out of the study at any time for any reason, without any prejudice. Written informed consents were obtained individually, as outline in the declaration of Helsinki. See methods section for more details.

## INSTITUTIONAL REVIEW BOARD STATEMENT

The ethical committee of the Università Campus Bio‐Medico di Roma (Rome – IT) approved the experimental phase I of the study entitled “Effect of quadrato MOtor Training On the BRAIN of healthy volunteers” (MOTO‐BRAIN, 09/14 PAR ComEt CBM). See methods section for more details.

## Supporting information


Table S1.

## Data Availability

All data generated during this study are included in this published article [and its supplementary information files]. Considering privacy issues related to clinical data, datasets analyzed during the current study are available from the corresponding author on reasonable request (e.g., submission of a formal project outline; requirements for co‐authorship; inclusion in the author byline).
